# The thermal ‘Buddha Board’—application of microstructured polyolefin films for variable thermal infrared transparency materials

**DOI:** 10.1098/rsfs.2023.0073

**Published:** 2024-06-07

**Authors:** Xiaoruo Sun, Mehbab Ali, Shima Jalali, Abolfazl Vaheb, Asad Asad, Patricia I. Dolez, James D. Hogan, Dan Sameoto

**Affiliations:** ^1^ Department of Mechanical Engineering, University of Alberta, Edmonton, Canada; ^2^ Department of Human Ecology, University of Alberta, Edmonton, Canada

**Keywords:** structural colouration, thermal infrared transmission, index-matching fluids, polyolefins, bioinspiration

## Abstract

In this study, we explore the innovative application of biological principles of scattering foams and structural colouration of white materials to manipulate the transmission properties of thermal infrared (IR) radiation, particularly within the 8–14 μm wavelength range in polyolefin materials. Inspired by the complex skin of organisms such as chameleons, which can dynamically change colour through structural alterations, as well as more mundane technologies such as Buddha Boards and magic water colouring books, we are developing methods to control thermal IR transmission using common thermoplastic materials that are semi-transparent to thermal IR radiation. Polyethylene and polypropylene, known for their versatility and cost-effectiveness, can be engineered into microstructured sheets with feature sizes spanning from 5 to 100 μm. By integrating these precisely moulded microstructures with index-matching fluids, specifically IR transparent oils, we achieve a reversible modification of the thermal transmission properties. This novel approach not only mimics the adaptive functionality of natural systems but also offers a practical and scalable solution for dynamic thermal management. Our results indicate a promising pathway for the development of new materials that can adapt their IR properties in real time, paving the way for smarter thermal management solutions via radiative emission/absorption.

## 1. Introduction

In the last few decades, the exploration of structural colour [1–4] and light-scattering material technologies [2,5–7] has emerged as a pivotal area of research, although primarily in the spectra of visible light and near-infrared (IR). This spectrum is critically important for the application of these bioinspired materials because natural surfaces provide a wealth of inspiration in how these wavelengths can be manipulated not through pigmentation but through selective or random scattering of light to produce brilliant colours, bright whites and dark blacks.

Recent advancements in the area of radiative cooling technologies have underscored the significance of designing materials capable of altering their reflectivity and absorptivity in a manner that is both rapid and reversible [7–9]. Switching reflectivity of visible and near-IR solar radiation is critical for innovative radiative heating and cooling applications as solutions adapt to changing weather conditions and times of the day. While occasionally this is done directly by switching surfaces entirely [8], other solutions have drawn inspiration from mechanisms akin to those observed in a Buddha Board, where water-induced changes lead to temporary changes in surface transparency [7,9–12]. Virtually all these solutions, however, are focused on transparency in the visible and near IR range to change the amount of solar energy heating up the solution, but future work could alter the transparency of surfaces so that thermal emission can be high or low [13] in addition to absorbing or reflecting incoming solar energy. It is the extension of switchable materials to the thermal IR spectrum (~8–14 µm) that corresponds to the atmospheric window that our work focuses on.

The development of materials with adjustable IR transparency aligns with the growing need for energy-efficient technologies. Given the global push towards sustainability, these materials can play a critical role in reducing energy consumption in various sectors, including building insulation and waste heat recovery [6–8,10,12,14–17]. The challenge lies in designing materials that not only respond swiftly and reversibly to external stimuli but also maintain their structural integrity and optical properties under varying environmental conditions. Also, of critical importance to scalability is to make these materials as inexpensive and durable as possible so that they may be deployed at scale in an inexpensive way.

In the study of light scattering by particles [18], the relative size of the particles compared with the wavelength of incident light critically determines the applicable scattering theory. For particle sizes *d* much smaller than the wavelength λ ( $d<\frac{\lambda }{10}$ ), Rayleigh scattering predominates, characterized by a strong wavelength dependence and is responsible for phenomena like the blue sky. As particle sizes become comparable to the wavelength Mie scattering becomes relevant. This theory comprehensively addresses a wide range of particle sizes but is particularly pertinent in this intermediate regime, where neither Rayleigh scattering nor geometric optics provide accurate descriptions. In the context of designing microstructured materials for scattering or reflecting thermal IR light, where the wavelengths are typically in the micrometre range, Mie scattering assumes a significant role. This is because the particle sizes in these materials often fall within the Mie scattering domain, necessitating its application for accurate modelling and understanding of their optical properties. For much larger particles or features ( d>10λ ), geometric or optical scattering principles, which consider light as rays, are more appropriate. For structural colouration in nature, photonic crystals that selectively absorb or reflect specific wavelengths will have highly ordered nanostructures smaller than the wavelength of visible light [1,4]. Because our area of interest is thermal wavelengths on the order of 10 µm, it is possible to get high scattering through features on the micron scale, which may be produced through a variety of processes, including lithography, melt blowing, electrospinning and others.

## 2. Manufacturing and test of thermal scattering materials

**Figure 1.**
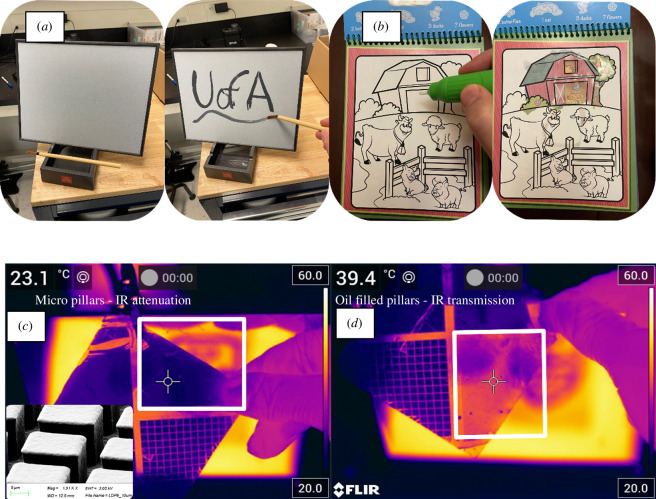
(*a,b*) Two examples of commercial products using index-matching fluid (water) to enhance the transparency of a microstructured material to show colour underneath. In the case of (*a*), a Buddha Board allows temporary art from the dark backing being visible when wet. In (*b*), a colourful picture is revealed after the board is wet with water. (*c*) shows a microstructured PE sheet with 13 µm thick pillars attenuating IR transmission. (*d*) shows an example of the same microstructured PE sheet becoming thermally transparent when wetted with mineral oil.

When disorder occurs in these nanostructures, a white appearance is achieved when uniform, non-specular reflection happens for all visible wavelengths [1,14,15]. A similar scattering phenomenon can also happen in clouds, snow and smoke where much larger particles can scatter light and result in a white appearance but these larger particles compared with visible wavelengths are much less efficient at scattering and thick layers are necessary before high scattering occurs [18]. This white, opaque appearance goes away, however, if snow is compressed into solid ice or is filled with water in a slush because the interfaces between dissimilar materials of different refractive indices are eliminated. Commercial products such as Buddha Boards and magic colouring books, also take advantage of this phenomenon. These products make use of a highly structured surface that is normally opaque/white but becomes highly transparent to visible light when water is applied and pores are filled with index-matching fluid as shown in figure 1*a,b*. What is new in our work is the demonstration that the exact same phenomena can be applied to thermally transparent materials and liquids primarily based on polyolefin chemistry (figure 1*c,d*). These molecules are relatively simple and have few types of molecular bonds that resonate and therefore absorb/emit in wavelengths corresponding to the atmospheric thermal window of interest, and can be considered thermally transparent in this range [19]. Polyethylene (PE) is, in general, the best performer, but polypropylene (PP) and a few other candidates can also work. Oils based on similar molecules, such as alkanes, are effectively short-chain polyolefins and were selected as index-matching fluids.

**Figure 2.**
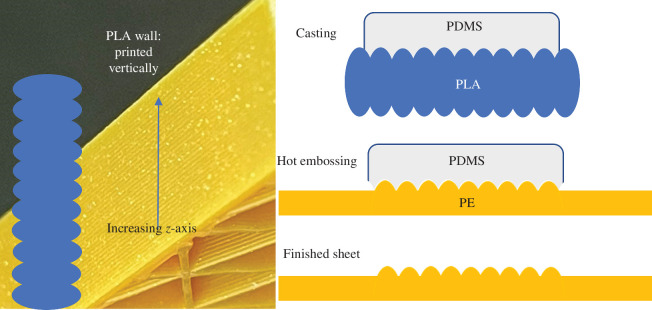
Use of the sidewalls for three-dimensional printed thermoplastic features as a scattering surface for thermal IR.

**Figure 3.**
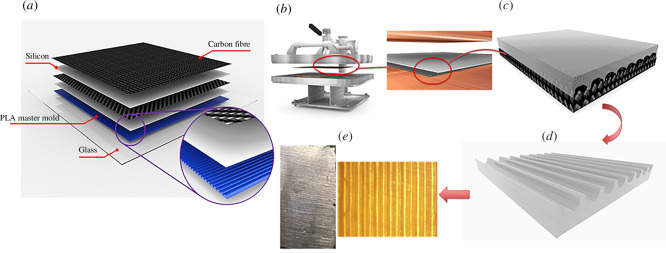
(*a*) Schematic of layer contributions in silicone mould fabrication; (*b*) hot press to apply force and pressure between pure PE and structured silicone mould; (*c*) final structured PE replicated on the silicone; (*d*) final structured PEs; and (*e*) PE lens-structured cylindrical lens structure for high scattering and low attenuation of IR.

**Figure 4.**
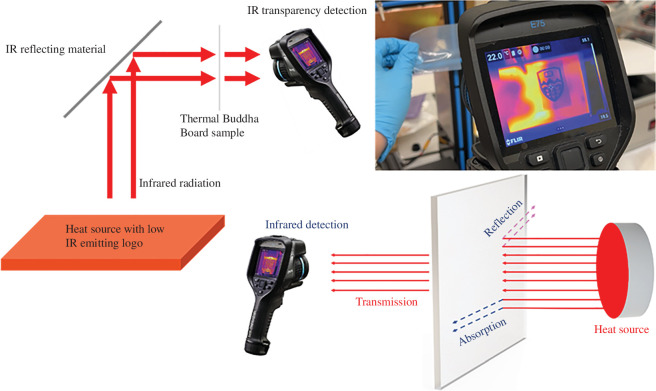
Thermal transmission tests for viewing apparent temperature and degrees of scattering in thermal IR. IR reflective surface was a solid copper sheet 0.025′ in thickness.

Testing of thermally variable materials was divided into two strategies: (i) randomly micro/nanostructured polyolefins from a variety of commercial sources and (ii) deterministically patterned solid sheets using soft lithography and thermal moulding. Randomly structured fabrics of polyolefin can be available for a variety of purposes, including melt blown PP as used in N95 and KN95 masks [20], Tyvek HDPE fabric [21] and nanoporous PE used in lithium-ion batteries [22]. Micromoulding of LDPE or HDPE was done with silicone rubber moulds from original SU-8 templates (see electronic supplementary material, SI) or vertical walls from three-dimensional printed moulds (figures 2 and 3) and could be done with a single material or on a backing of ultra-high molecular weight PE (UHMWPE) for more uniform layer thickness and enhanced durability. UHMWPE was too high viscosity as a melt to uniformly mould in silicone rubber. The focus on visible and IR transmission led to tests relying on an FLIR E75 thermal camera to compare the apparent temperatures through different materials. All structured materials were at ambient temperatures (~21°C) when tested in the lab. Two different setups were completed using thermal IR sources as shown in figure 4. The systems were used to prevent conductive heating of structured samples during tests.

Based on the reported refractive indices of the polymers used, different liquids were chosen that had similar reported values. A comparison of properties is given in table 1.

**Table 1.** Reported refractive indices for materials used in these tests for visible wavelengths.

liquid and material	refractive index
acetone	1.36
isopropyl alcohol	1.38
dodecane	1.42
white mineral oil	1.47
PP	1.47
PE	1.51

Acetone and isopropyl alcohol were not expected to be very IR transparent based on their FTIR spectra but dodecane was selected as an index-matching fluid because it is in effect a very short-chain PE, with a molecular formula of CH_3_(CH_2_)_10_CH_3_, remains liquid between −10°C and 214°C and evaporates relatively slowly, so as not to introduce evaporative cooling [23]. It was anticipated that dodecane’s thermal transparency properties would be very close to those of either PE or PP. White mineral oil (purchased as UltraPro Food grade from Amazon.ca), on the other hand, is a complex mixture of refined saturated and aromatic hydrocarbons as a byproduct of petroleum refining. Its exact composition is unknown, but it is very inexpensive (~$22/l versus ~$500/l) in comparison to dodecane in Canadian prices in 2023. It had the best thermal transparency of the various inexpensive oils tested in initial prototyping (including vegetable oils, silicone oils and olive oil—all far less transparent to thermal IR).

**Figure 5.**
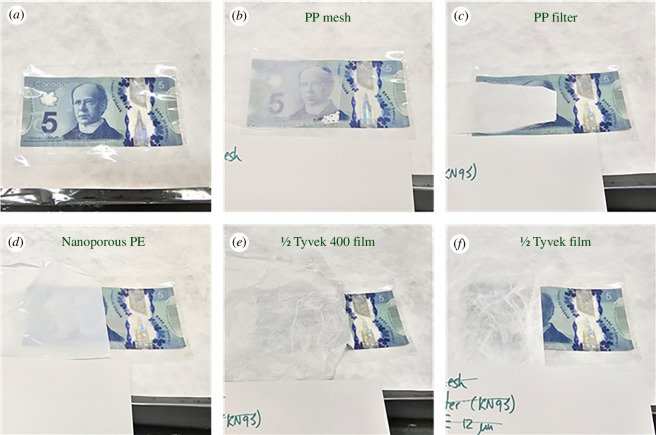
Canadian $5 bill sealed in a transparent PP pouch with different scattering polyolefin fabrics and films on top for visual comparison of opacity. (*a*) $5 bill under a transparent PP film. (*b*) bill observed through PP mesh, (*c*) bill observed through PP filter, (*d*) bill observed through 12 µm thick PE film, (*e*) bill observed through a delaminated Tyvek 400 fabric and (*f*) bill observed through a delaminated Tyvek fabric.

A comparison of transparency in the visible spectrum was completed by looking directly at commercial polymer samples laid over the top of a Canadian $5 bill for qualitative measurements. Tyvek fabrics were delaminated into two halves to reduce their overall thickness by tearing an edge and peeling the top and bottom portions apart. The PP mesh was the outer surface of a KN95 mask, and the filter was the inner portion of finer melt-blown PP from the same mask. Pictures of all materials before wetting with any liquid are shown in figure 5.

**Figure 6.**
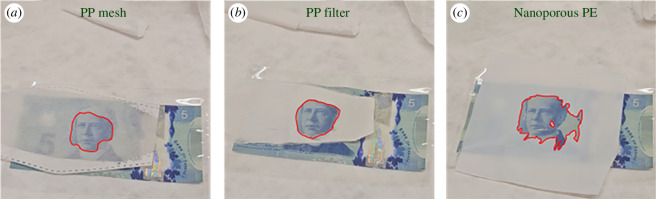
Optical pictures of transparency changes with the addition of mineral oil onto different fabrics and films (outline of the interface highlighted in red). (*a*) shows a PP mesh with thicker fibres and some thermally bonded areas, (*b*) shows a PP filter with much finer fibres and thicker structures for more scattering. (*c*) shows a nanoporous PE membrane. The PP filter which has no closed pores and high matching between mineral oil and PP index of refraction shows the largest clarity difference.

As expected, the fabrics all made the viewing of structures immediately behind them very difficult, but the highest scattering was found to be with the PP filter which was thickest and had the finest fibre features, and the Tyvek fabrics, which were approximately 3‰ thick. The nanoporous PE, which was a solid film with pores was not as scattering as Tyvek but was only ~0.5‰ thick, making it quite a bit better at scattering if compared with the thickness of material used. When different liquids were placed on the materials, there was a trend of improved optical transmission as the liquid index of refraction got closer to that of the fabric material (electronic supplementary material, figure S2). For the mineral oil, the match with PP of the index of refraction was extremely close and it was the closest of the liquids to PE, which did not an exact match for any liquid. The resulting clarity of the PP filter when wet with mineral oil was therefore the most dramatic of any combination, although the nanoporous PE was also substantially changed owing to the very small thickness of the material. Optical images of these wetted samples are shown in figure 6.

A similar technique was applied to these materials in the thermal IR regime, with the exception that the ‘visible’ features were separated by a significant distance as shown in figure 2. The reason for this is that all materials emit thermal energy at temperatures above zero Kelvin with an energy emission proportional to the fourth power of temperature. As a result, when imaging with a thermal camera, the entire field of view is constantly being compared as relative temperatures and as if emissivity values are equal for all objects. Our goal then is to compare the effective temperature that can be seen through a microstructured sheet with and without index-matching fluids and determine the apparent temperature of the area under view while knowing that the temperature of the sheet is always at ambient temperatures.

**Figure 7.**
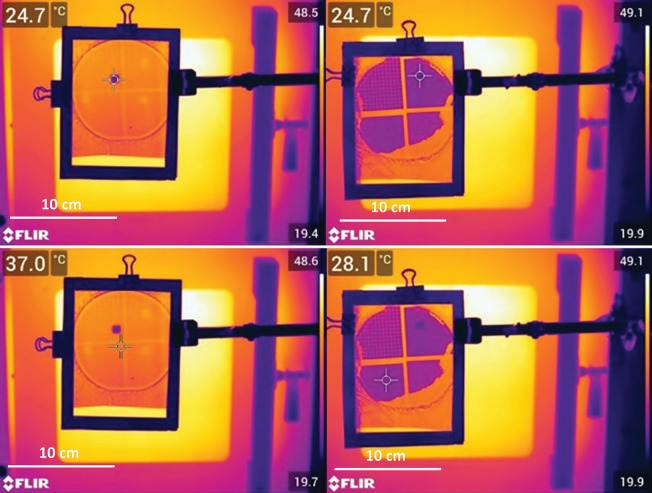
Structured PE sheets with square posts that are either ~2 µm (left) or ~10 µm tall moulded from LDPE on top of a 100 µm thick UHMWPE sheet. A single metallic square taped to a hot oven is used as a feature to determine approximate scattering and attenuation—virtually no thermal scattering, but high attenuation is achieved with these rectangular and vertical pillars.

**Figure 8.**
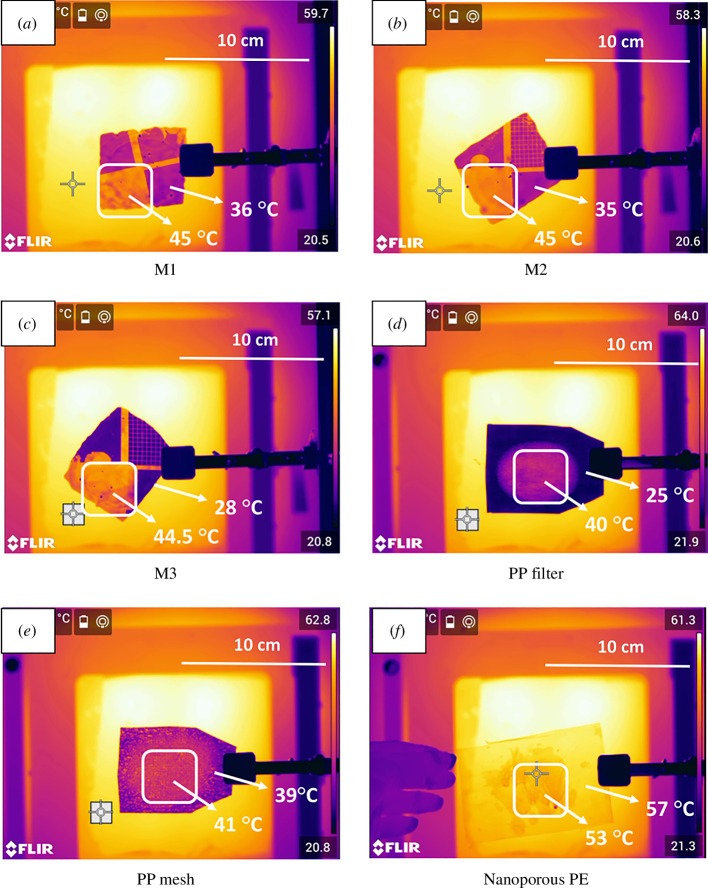
Thermal Buddha Board effects on different candidate materials. M1–M3 are square pillars moulded on PE with either 10 (*m1*, *m2*) or 13 (*m3*) µm heights. The largest difference in thermal attenuation is seen for M3 when wetted and the pillar height has a more substantial impact than width. The nanoporous PE decreases in thermal transparency when mineral oil is added because the overall thickness is increased compared with the initial 12 µm and thermal transparency is already extremely high because the pore size is substantially smaller than IR wavelength.

To get more knowledge on the general applicability of the microstructure feature effects on the thermal IR transmission, PE sheets moulded with square microfeatures were produced. These had regular spacing and feature sizes and were produced with a variety of sizes as noted in table 2. Surprisingly, for the range of samples tested, the feature sizes were far less important than the height of the structures. While reaching a maximum height of only approximately 13 µm, these sheets could attenuate IR light to the equivalent of solid PE thicknesses many 100 s of µm thick. Attractively, these materials only have two air-polymer interfaces so are more easily modelled for deterministic properties [24]. A comparison of features 2 and 10 µm tall but otherwise similar in structure is shown in figure 7 and the combination of mineral oil on different Buddha Board candidates is shown in figure 8. The results demonstrate that the largest difference in thermal transparency comes from the rectangular pillars 13 µm tall when wetted with mineral oil. This microstructure design provides very high thermal attenuation with minimal scattering. The PP filter has extremely high attenuation and scattering while the nanoporous PE has minimal scattering or attenuation in thermal IR despite being visibly opaque. The lesson is that the scale of the scattering features is critical for the wavelengths used and can be deterministically designed in future versions to optimize for specific thermal or visible transparency.

**Table 2.** Dimensions of micropillars for lithographically defined thermal scattering surfaces.

mask 1	post dimensions (µm)	height (µm)
m1	6 × 6–9 × 9	10
m2	10 × 10 – 13 × 13	10
m3	10 × 10 – 13 × 13	13

**Figure 9.**
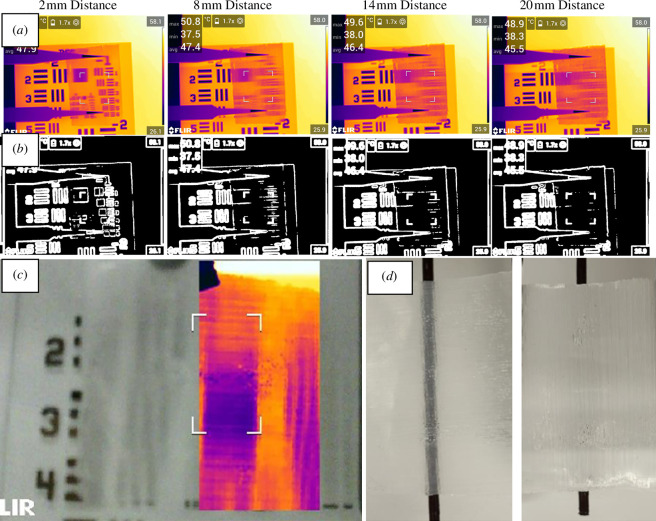
*(a*) Thermal scattering and low attenuation effect of cylindrical lens structures with 40 µm pitch manufactured from PE. (*b*) shows an edge detection algorithm applied to the thermal images [13]. As different distances from the hot source are tested, larger features lose their ability to be resolved by the thermal camera. At this scale of features, traditional optical design may be appropriate. (*c*) shows the distinctive directional blurring of features similar to Lubor’s lens in both thermal and IR simultaneously and (*d*) shows optical images of the same effect.

One final test of interest was that of larger structures than can normally be produced by lithography, and these were cylindrical lens arrays manufactured from PE using a three-dimensional printed master mould. These were designed with the goal of maximum thermal scattering and minimum attenuation. The features were all at least double the wavelength of IR light (minimum 40 µm pitch) and were round-shaped lenses rather than rectangular. The general performance demonstrated that the rounded features could scatter thermal and IR light very effectively and showed similar behaviour to the magic trick Lubor’s lens, where blurring of features in one direction is extreme yet perpendicular features to the lens alignment are easily viewed. Attenuation was negligibly changed compared with features designed closer to the Mie scattering size regime which can be a useful feature as in future their thermal wavelengths may be desirably focused or directed using features like Fresnel lenses. These features and their scattering behaviour in thermal and visible spectrums are shown in figure 9.

## 3. Conclusion

We have successfully demonstrated for the first time a proof-of-concept thermal ‘Buddha Board’ that takes inspiration from natural and synthetic scattering surfaces and uses index-matching fluid to make them transparent to thermal IR light. Using some of the most common and low-cost industrial polymers demonstrates that these techniques could be applied at a large scale with minimal change to industrial manufacturing processes, and future work to integrate self-cleaning or anti-wetting properties may be a high priority to ensure that maximum thermal transparency is maintained. We hope that this solution can make its way into a variety of next-generation thermal management solutions.

## Data Availability

All data generated are included in the published paper and supplementary material [25].
